# A Study of English Learning Vocabulary Detection Based on Image Semantic Segmentation Fusion Network

**DOI:** 10.3389/fncom.2022.895680

**Published:** 2022-06-02

**Authors:** Leying Pan

**Affiliations:** School of International Studies, Zhejiang Business College, Hangzhou, China

**Keywords:** multi-modal discourse analysis, learning, image semantic, feature fusion, Deeplab V3+ network

## Abstract

College students learn words always under both teachers' and school administrators' control. Based on multi-modal discourse analysis theory, the analysis of English words under the synergy of different modalities, students improve the motivation and effectiveness of word learning, but there are still some problems, such as the lack of visual modal memory of pictures, incomplete word meanings, little interaction between users, and lack of resource expansion function. To this end, this paper proposes a stepped image semantic segmentation network structure based on multi-scale feature fusion and boundary optimization. The network aims at improving the accuracy of the network model, optimizing the spatial pooling pyramid module in Deeplab V3+ network, using a new activation function Funnel ReLU (FReLU) for vision tasks to replace the original non-linear activation function to obtain accuracy compensation, improving the overall image segmentation accuracy through accurate prediction of the boundaries of each class, reducing the intra-class error in the prediction results. The accuracy compensation is obtained by replacing the original linear activation function with FReLU. Experimental results on the Englishhnd dataset demonstrate that the improved network can achieve 96.35% accuracy for English characters with the same network parameters, training data and test data.

## Introduction

English Word Memory provides solutions for teaching English vocabulary in college. Some competitions have been held in Jiangsu alone with over 90 undergraduate and higher education institutions and over 200,000 students participating. Unlike the traditional way of learning by reading word books and memorizing words, the corpus-based English word platform brings together a variety of learning contents such as pronunciation, spelling and example sentences, and collaboratively uses media forms such as sound, image, text and color to generate dynamic vocabulary exercises, allowing students to learn word collocations and usage in the exercises, improving the efficiency of learning. In addition, the platform provides teachers with management and supervisory functions for vocabulary teaching (Liu et al., 2007; Liu, 2021).

Both the design and the use of English words contain a five-level system of multimodal discourse analysis theoretical framework. At the cultural and contextual levels, English Words users are divided into a teacher side and a student side (Dai et al., 2018; Yin, 2021). Because both teachers and students are in the same cultural context, the ideology and the structure of the subject matter are potentially the same, and the scope of the discourse is the same (Huang et al., 2019). The word content of English Words is designed from the textbook in which it is taught, and reflects the conceptual meaning and schematic meaning of words through word interpretation, usage, and example sentences in both English and Chinese. Through “check-in” and “ranking”, students and teachers can interact with each other and realize interpersonal meaning (Wu and Chen, 2020).

At the formal level, the different formal systems for achieving meaning include the “lexico-grammatical system of language” (Sung et al., 2016). The lexicon refers to the items that are already given meaning in their own right, while the grammar is a more complex system of structural rules for combining these items. Since one modality cannot fully express the meaning of communication. Other modal forms need to be used to enhance and complement the meaning. Chen Hsieh et al. (2017) classifies the relationship of multimodal discourse forms into two types-“complementary” and “non-complementary”. English words are dynamically generated based on a large-scale corpus of vocabulary exercises for students, and the non-reinforcing relationship between visual and auditory modalities is used to complement each other through word pronunciation identification, interpretation, and detailed example sentences, so that students can repeatedly compare and contrast in different contexts to promote learning through practice (Huang et al., 2011; Duman et al., 2015).

Image semantic segmentation, as a cornerstone technique in computer vision tasks, is different from target detection and image classification in that each pixel in an image is assigned a predefined label indicating its semantic class to achieve the task of pixel-level classification (Saalbach et al., 2009; Chen et al., 2021). Specifically, image semantic segmentation is the process of distinguishing at the pixel level exactly what and where the target object is in an image, i.e., first detecting the target in the image, then depicting the outline between each individual and the scene, and finally classifying them by assigning a color to things that belong to the same class (Lyu et al., 2018; Zhang et al., 2020).

In recent years, with the development of deep learning technology in computer vision, image semantic segmentation has been widely used in autonomous driving, intelligent medical treatment, etc. (Sanonguthai, 2011). The intrinsic invariance of DCNN (Di Wu et al., 2021) can learn dense abstract features, which is much better than the performance of traditional systems designed based on sample features. However, existing semantic segmentation algorithms still suffer from intra-class semantic misidentification, small-scale object loss, and blurred segmentation boundaries. Therefore, capturing more feature information and optimizing for the target boundary are important research elements to improve the segmentation accuracy.

In 2016, Xue et al. (2018) proposed Deeplab V2 model based on Deeplab V1 network (Zhang et al., 2018), using inflated convolution instead of partial pooling operation for down sampling filter for feature extraction, and using spatial deterministic pyramid pooling (ASPP) module (Xie et al., 2018) for multi-scale feature extraction, In 2017, Deeplab V3 (Laufer, 2006) improved the ASPP module on the basis of V2 network to form an end-to-end network structure, and eliminated the CRF boundary optimization module. In the field of semantic segmentation, the network structure usually adopts the codec-decoder structure; except for Deeplab V3+, almost all of the above mentioned algorithms do not consider using the effective decoder module, or only use the codec-symmetric structure with a single structure, which fails to effectively fuse the high-level semantic information and the low-level spatial information across layers in the up sampling process, and loses the important pixel information of the feature map.

### Related Work

Through literature combing, we found that the current research on adaptation of English learning supported by artificial intelligence is mainly about the design and development of adaptive, wisdom-adapted related learning systems for students, and there is no literature on learning adaptation from the students' perspective. Since, learning adaptability is related to learning performance, learning quality, etc. Therefore, the literature is extended to study “learning performance,” “learning effectiveness,” “learning quality,” “teaching effectiveness,” and “teaching quality” related to artificial intelligence-supported learning. “Teaching quality,” etc. (Duman et al., 2015; Xue et al., 2018; Zhou et al., 2020). A review of the literature shows that the research focuses on two aspects of speaking and composition, and specific teaching practices of AI English learning tools are mainly educational APPs and intelligent online systems. In speaking training and assessment, Gorman's “English Fun Dubbing” has stimulated students' interest and confidence in speaking learning, and thus improved students' English learning petrifaction (Hessamy and Ghaderi, 2014). Liu et al. (2021) study came to a similar conclusion that although a small number of students were not very active, most of them were able to accept the learning mode of using English Fun Voiceover to learn speaking, and there was a significant difference between English majors and non-English majors in their willingness to use English APPs for listening and speaking. In terms of smart writing, the smart writing system criterion significantly improved the quality of students' writing in Attali, which found that the number of student essay revisions was positively correlated with improved scores, but 70% of the students in that study lacked confidence or interest in the system (Cameron, 2002), and if teachers do not approve of the smart writing system, then students also If teachers do not approve of the intelligent writing system, then students will also lack motivation to use it consistently. Gu and Zhang (2020) pointed out that the intelligent composition review system can help students develop the habit of repeated revision, but it should not be It is still necessary to have teacher guidance. Zhang and Liu (2021) study found that course assessment mechanisms, students' vocabulary levels, their perceptions of the feedback from the intelligent writing system, and the quality of the intelligent system itself affect students' use, and that students' motivation to learn English affects their learning outcomes in the automatic evaluation feedback system. Zhao et al. (2017) concluded that at the individual level, students' familiarity with computers, online learning experience, existing language ability and writing level, and learning autonomy bring about different usage effects in the collaborative artificial intelligence system, but there are no related research topics.

## Proposed Algorithm

### Deeplab V3+ Network Architecture

The Deeplab V3+ network architecture is the latest generation of semantic segmentation network framework in the Deeplab series proposed by Google Labs, with superior performance on multiple datasets. Or Xception as the backbone network, using a data normalization (BN) layer to prevent training overfitting (Liu et al., 2020), and adding a decoder network component to build an end-to-end coder-decoder network model.

**Figure 1:**
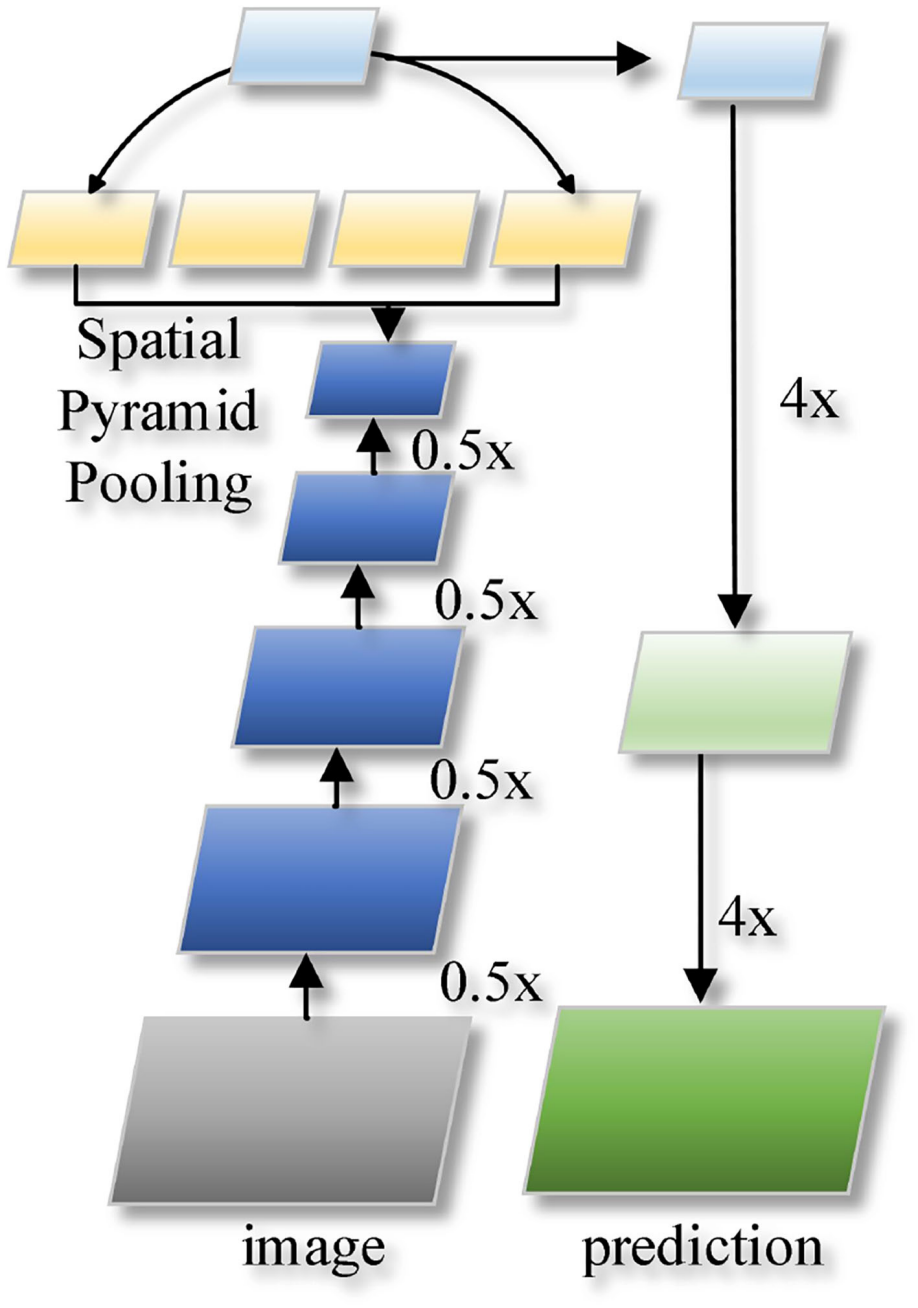
DeeplabV3+ network structure.

The structure of DeeplabV3+ network is shown in Figure 1. The input image is passed through a neural network with an inflated convolution to reduce the number of down sampling while ensuring a large perceptual field, and the high-level semantic information and low-level spatial information are extracted separately. The number of channels is adjusted using the convolution operation, and the bilinear FOE (Tian et al., 2019) quadruple up sampling is used to fuse the low-level spatial information with the adjusted number of channels across the layers, and the quadruple up sampling restores the original image resolution and spatial details.

### Improved Stepwise Deeplab V3+ Network

**Figure 2:**
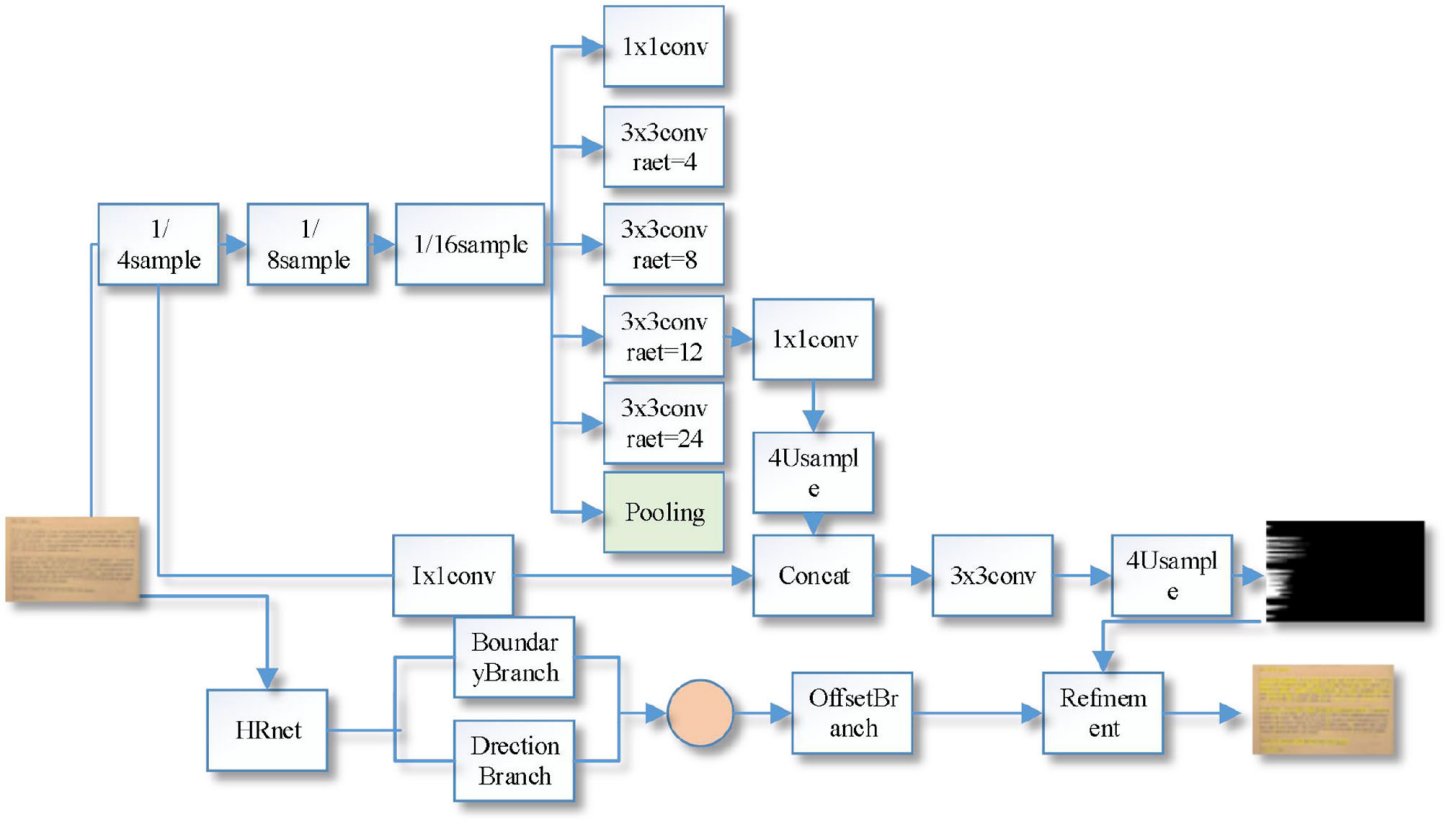
Improved ladder-type DeeplabV3+ network structure.

Compared with the Deeplab V3+ network, the large scale target prediction is more prone to the problem of missing small scale targets and rough category boundaries. The improved Deeplab V3+ network is shown in Figure 2, which is based on ResNet-101 (Xie et al., 2019) as the backbone network, including encoder, decoder, and optimizer.

DeeplabV3+ network uses V3 model as encoder, and continues to use the original expansion convolution of V3 model ASPP module with expansion rate of 6, 12, and 18, while the feature map resolution decreases as CNN extracts the image feature information. Considering that when extracting low-resolution features, the expansion convolution of 4 and 8 can better capture the details of small-scale targets than the expansion convolution of 6, and when segmenting large-scale targets, it is necessary to obtain a larger sensory field, and the expansion convolution of 24 has a larger sensory field than the expansion convolution of 18, which is more favorable when segmenting large-scale targets. The ASPP parameters proposed in this paper are compared with the ASPP modules (6, 12, 18) provided by the V3+ model, and the proposed parameters are better than the original ones.

The original Deeplab V3+ model only designs a simple decoder, and the decoder mainly handles high and low-level feature map fusion operations; when performing feature map cross-layer fusion, considering that the 1/4 times downsampled feature map of the ResNet101 network contains rich low-level spatial information, while the 1/16 feature map generated by the encoder ASPP module contains rich high-level Therefore, in the fusion of feature maps, it is necessary to resize the high-level feature map generated by the ASPP module to the low-level feature map generated by the backbone network, so the 1/16th feature map generated by the encoder ASPP module is upsampled 4 times and then fused with the 1/4th feature map generated by the backbone network. Then convolution and upsampling operations are performed to generate the prediction result map; the ReLU activation function is used in the original codec network for non-linear activation, the reliability of the ReLU activation function has been recognized in the field of deep learning, but it lacks pixel-level modeling capability in computer vision tasks, so this paper uses the two-dimensional visual activation function FReLU to replace the ReLU activation function in the codec to obtain accuracy compensation (Shin et al., 2016).

### Encoder Optimization

**Figure 3:**
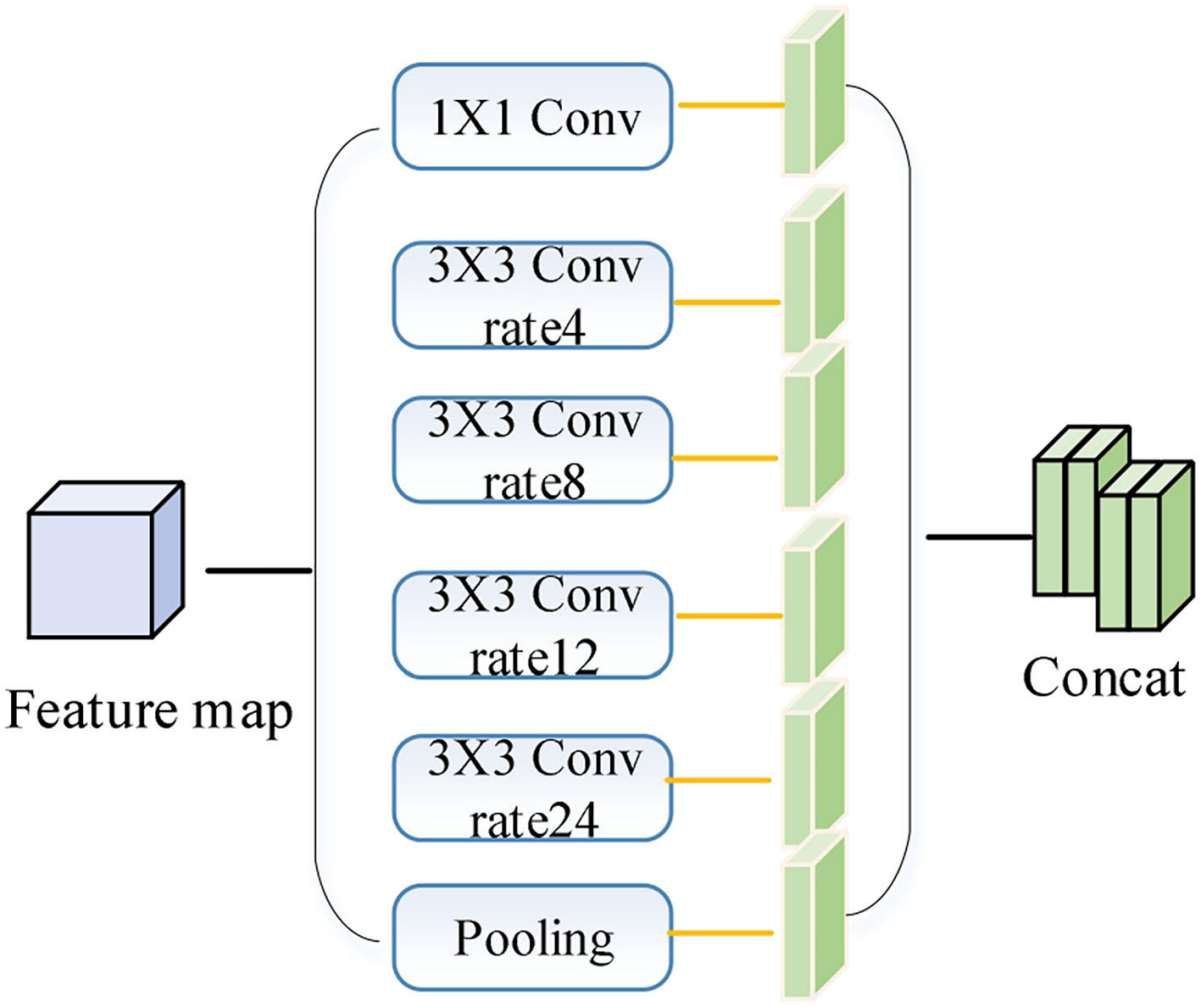
Improved ASPP module.

The ASPP module passes the input feature map evenly through different expansion rates of expansion convolution and global average pooling layers. The smaller expansion rate is more effective in segmenting small-scale targets; the larger expansion rate is more effective in segmenting large targets. The ASPP module in the encoder is improved as shown in Figure 3. The 1/16 feature maps generated by the backbone network are put into the 1 × 1 convolution, the expanded convolution with 4, 8, 12, and 24 expansion rates, and the global average pooling layer to generate 6 1/16-size feature maps with 256 channels, and the 6 feature maps are stitched together in the channel dimension to generate the ASPP module feature maps. The ASPP module feature maps are stitched together in the channel dimension to generate ASPP module feature maps, which can better extract multi-scale image features and improve the segmentation capability of the network for different scales of objects.

### Code-and-Decoder Modeling Capability Optimization

In deep learning, CNN have good performance superiority in processing visual tasks. Non-linear activation function is a necessary component of CNN to provide good nonlinear modeling capability (Shin et al., 2016). Nowadays, the main common activation functions are ReLU and its evolved PReLU.


(1) $$\begin{array}{l} ReLU(x)=\{\begin{array}{ll} x & \text{ if }x>0\\ 0 & \text{ if }x\leq 0 \end{array}\\ PReLU(x)=\{\begin{array}{ll} x_{i}, & \text{ if }x_{i}>0\\ a_{i}x_{i} & \text{ if }x_{i}\leq 0 \end{array}) \end{array}$$

ReLU as the most commonly used activation function, when the input is greater than zero, for the linear part of the function. However, when the input is less than zero, the function is adjusted by artificially setting the zero value. Therefore, there is a dead zone of activation, which leads to the poor robustness of the activation function during training, and the problem of “necrosis” of neurons when facing large gradient input. The gradient value is zero.

PReLU adds a linear activation part to the input less than zero by introducing a random parameter a that varies with the data computation. The above activation functions have been applied in various fields of deep learning with proven reliability. However, in the field of computer vision, these activation functions are unable to extract finer pixel-level spatial modeling capabilities, so the semantic segmentation network FReLU, a visual task activation function proposed by Shin et al. (2016) and Zhang et al. (2019), is used to compensate for the accuracy and obtain richer spatial contextual semantic information.

**Figure 4:**
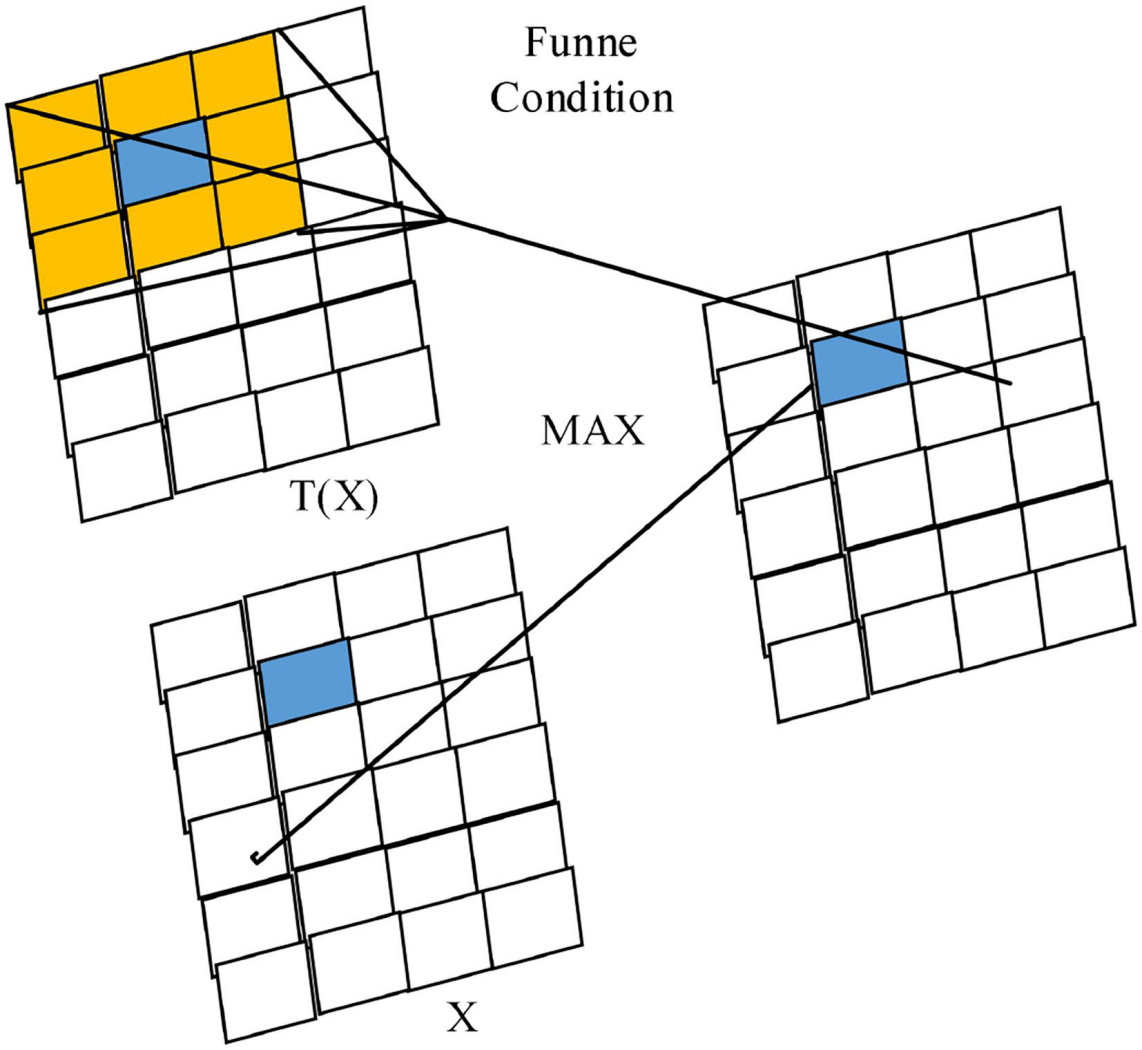
Two-dimensional FReLU activation function with funnel condition.

FReLU is a two-dimensional funnel-like activation function proposed specifically for computer vision tasks, which is expanded to two dimensions by adding the funnel condition T(X) to the one-dimensional ReLU activation function (as shown in Figure 4), introducing only a small amount of computation and overfitting risk to improve the vision task with spatially insensitive information in the activation network, with the expression:


(2) $$\begin{array}{l} f\left(x_{c,i,j}\right)=max\left(x_{c,i,j},\;\text{T}\left(x_{c,i,j}\right)\right)\\ \text{T}\left(x_{c,i,j}\right){=}_{c,i,j}^{\omega }\cdot p_{c}^{\omega } \end{array}$$

where *x*_*c, i, j*_ is the two-dimensional spatial location of the cth channel non-linear activation function *f*(.) and function *T*(.) is the functor condition; $x_{c,i,j}^{\omega }$ is the parametric pooling window on *x*_*c, i, j*_; $p_{c}^{\omega }$ is the shared coefficient on the common channel; (.) is the dot product operation.

Its funnel condition is a square sliding window with preset parameters, which is realized by deep separable convolution and data normalization (BN), which can enhance the spatial dependence between pixel and pixel kweek, activate spatially insensitive information still while obtaining rich spatial context information, and improve the pixel-level spatial modeling capability. The graphical depiction of the funnel condition pixel-level modeling capability is shown in Figure 4; only a small number of parameters are introduced, introducing very little complexity. Considering the fact that in natural objects, besides vertical and horizontal directions, diagonal and circular arcs are also common, the pixel spatial information extracted by different activation layers is represented by squares of different sizes, and the diagonal and circular arc activation domains are formed by extreme approximation thinking to avoid the lack of modeling capability caused by using only the usual horizontal and vertical activation domains (Radwan et al., 2016).

## Method Implementation

### Data Pre-processing

The effect of this paper's model in English semantic analysis is verified. English is composed of letters, and compared with other languages, there are only 26 letters in English, and the form changes are relatively simple, so English characters can be recognized directly by neural networks.

**Figure 5:**
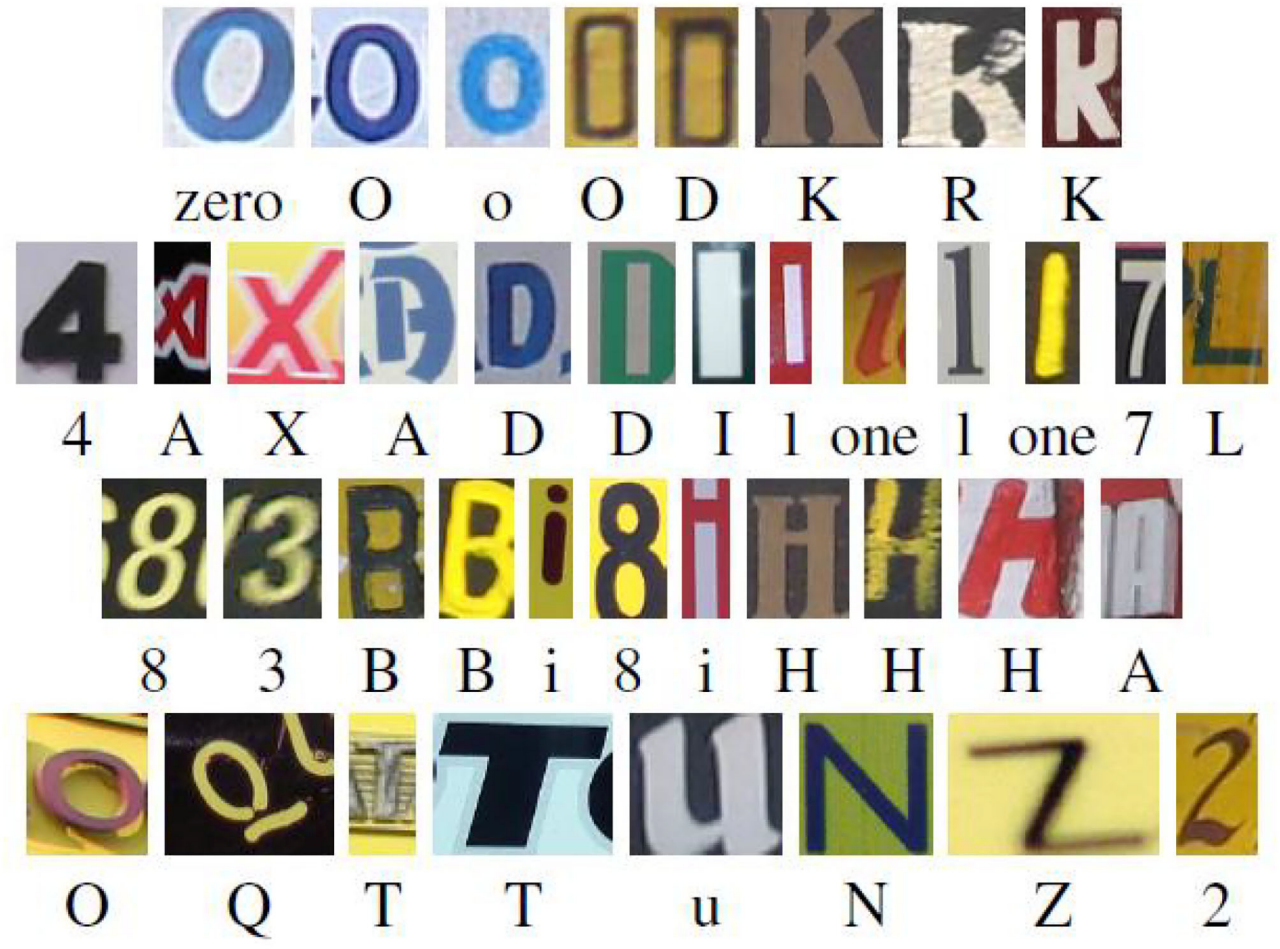
Englishhnd dataset.

The dataset used in this paper is Englishhnd (Saghezchi et al., 2013), in which the symbols used in English and Kannada are included. The English language includes: Latin characters (excluding accent marks) and Arabic numerals, and the dataset includes 64 classes (0–9, a–z, A–Z) of characters. Among them, there are 7,705 characters from natural images, 3,410 characters input by computer handwriting board, and 62,992 characters merged by computer font. Some of their characters, as shown in Figure 5.

**Figure 6:**
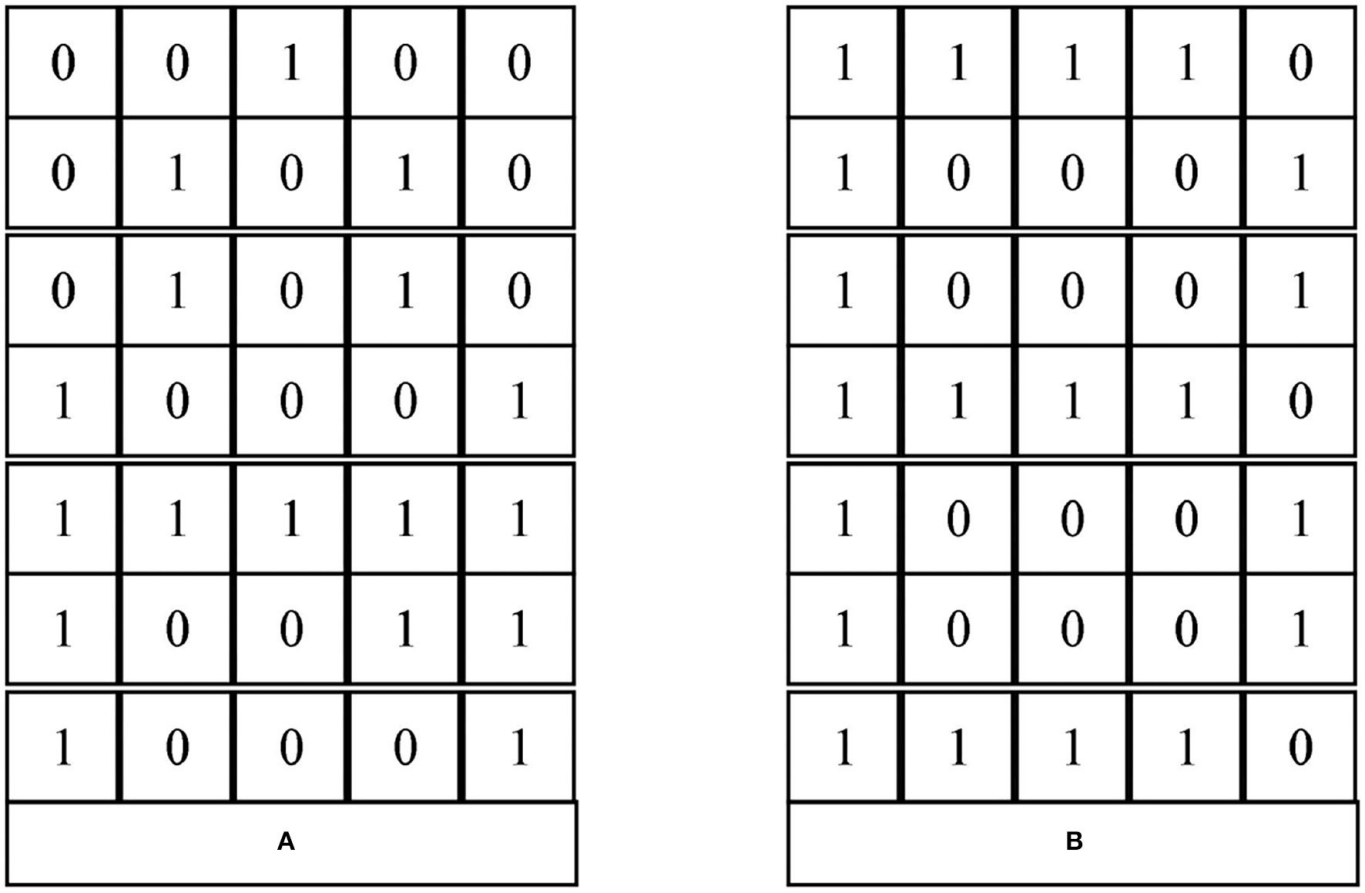
Representation of the letters A and B.

Since this paper only recognizes English characters, firstly, the characters corresponding to “0~9” in the data set are screened out. Second, each English letter is digitally represented as a 7 × 5 squares, as shown in Figure 6.

Figure 6 gives the digital representation of the capital letters A and B. The part of the letter with data is represented by 1 and the part without data is represented by 0. For different 52 letters (including case) there are 52 different representations. Then, according to the order from rows to columns, we can get 3 vectors of dimension 35 for different letters. “A” and “B” is represented as follows:

A = [0 0 1 0 0 0 1 0 1 0 0 1 0 1 0 1 0 0 0 1 1 1 1 1
1 1 0 0 0 1 1 0 0 0 1]
B = [1 1 1 1 0 1 0 0 0 1 1 0 0 0 1 1 1 1 1 0 1 0 0 0
1 1 0 0 0 1 1 1 1 1 0]

After digitizing the characters, the captured images are often disturbed by noise due to the actual English character recognition. Therefore, in order to simulate the actual application scenario, this paper superimposes noisy data on Englishhnd. The operation of adding noise can be implemented by the rand function in python software.

### Simulation Results

In order to better evaluate the performance of the RBF network, a BP neural network is used in the paper for comparative simulation tests. In order to ensure the consistency of time and space complexity during the training of the two networks, the parameters of the two networks, as shown in Table 1.

**Table 1:** Network parameters.

**Parameter name**	**Parameter value**
Number of hidden layers	1
Number of hidden units	500
Enter the number of nodes	35
Number of output nodes	52
Target error	0.0001
Maximum training times	40

**Figure 7:**
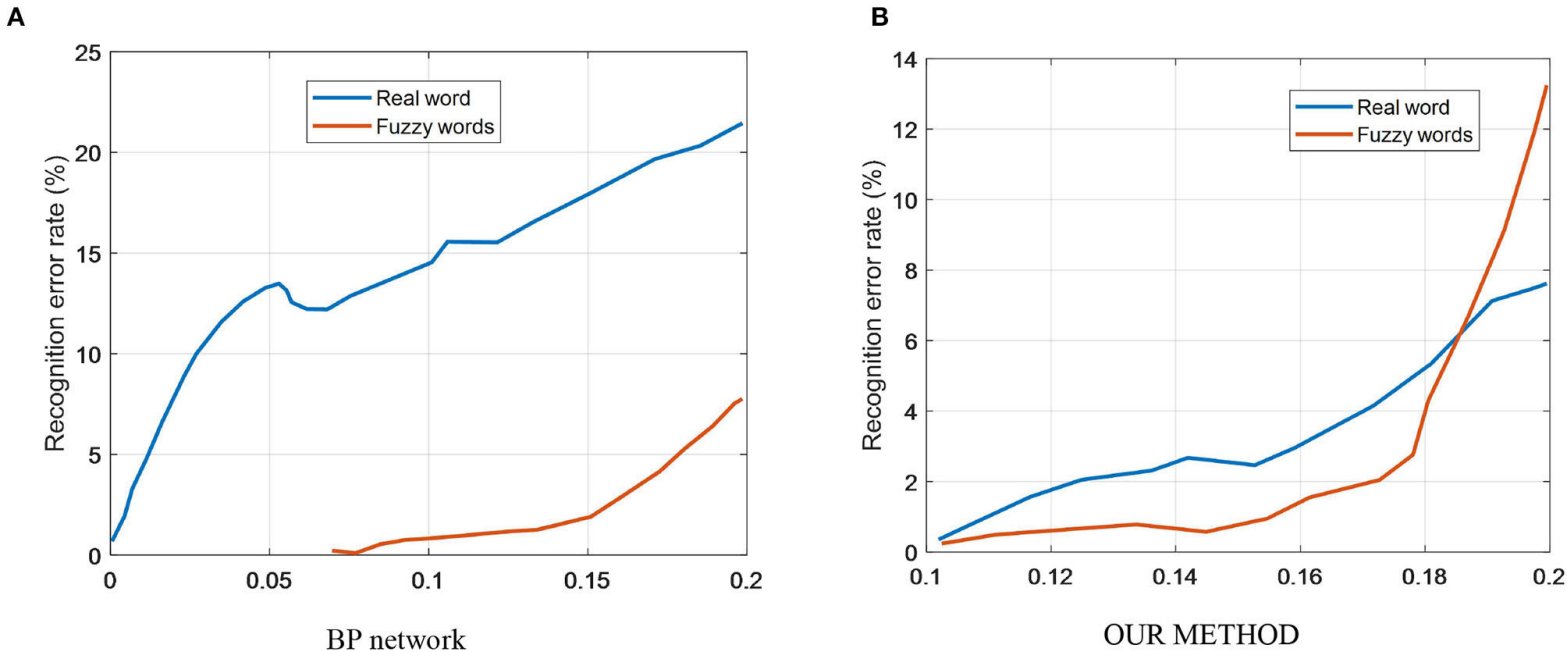
Relationship between word vector dimensionality and F1, training time. **(A)** BP network. **(B)** Our method.

In Figure 7, the solid line shows the change in the error rate at test with the test dataset after adding noise after training with the ideal signal; the dashed line shows the change in the error rate after using the noise-added signal.

As can be seen from the solid line in Figure 7A, the BP network is trained using the ideal signal without noise, and the error rate of the network recognition increases more when the test dataset is noise-added. The dashed line in Figure 7A shows that the network is less affected by noise in the test when trained using characters with noise-added signals. Therefore, the BP network is more disturbed by image noise, and this network has better recognition accuracy only when the test set is not noise-added.

The solid line in Figure 7B shows that when the our model is trained with noiseless data, the recognition error rate of the network only changes significantly after the mean value of noise exceeds 0.1 for the test data.

As can be seen from the dashed line in Figure 7B, when training with noisy data, the performance of the network also deteriorates after the mean value of noise exceeds 0.1 for the test data. Since the dashed line follows basically the same trend as the solid line, the our model is less disturbed by noise when performing character recognition and has stronger noise immunity compared to the BP neural network.

Table 2 gives the test results of BP and our models with a noise level of 0.1 for the test set after adding noise to the training data.

**Table 2:** Dataset parameters.

	**BP network**	**Our model**
Recognition accuracy	88.56%	96.35%
AUC	0.72	0.89

As can be seen from Table 2, with the same network parameters, training data and test data, the recognition accuracy of our model for English characters can reach 96.35%, which is 7.79% higher than that of BP network (88.56%); the AUC of our model reaches 0.89, which is closer to 1 than that of BP network (0.72).

**Figure 8:**
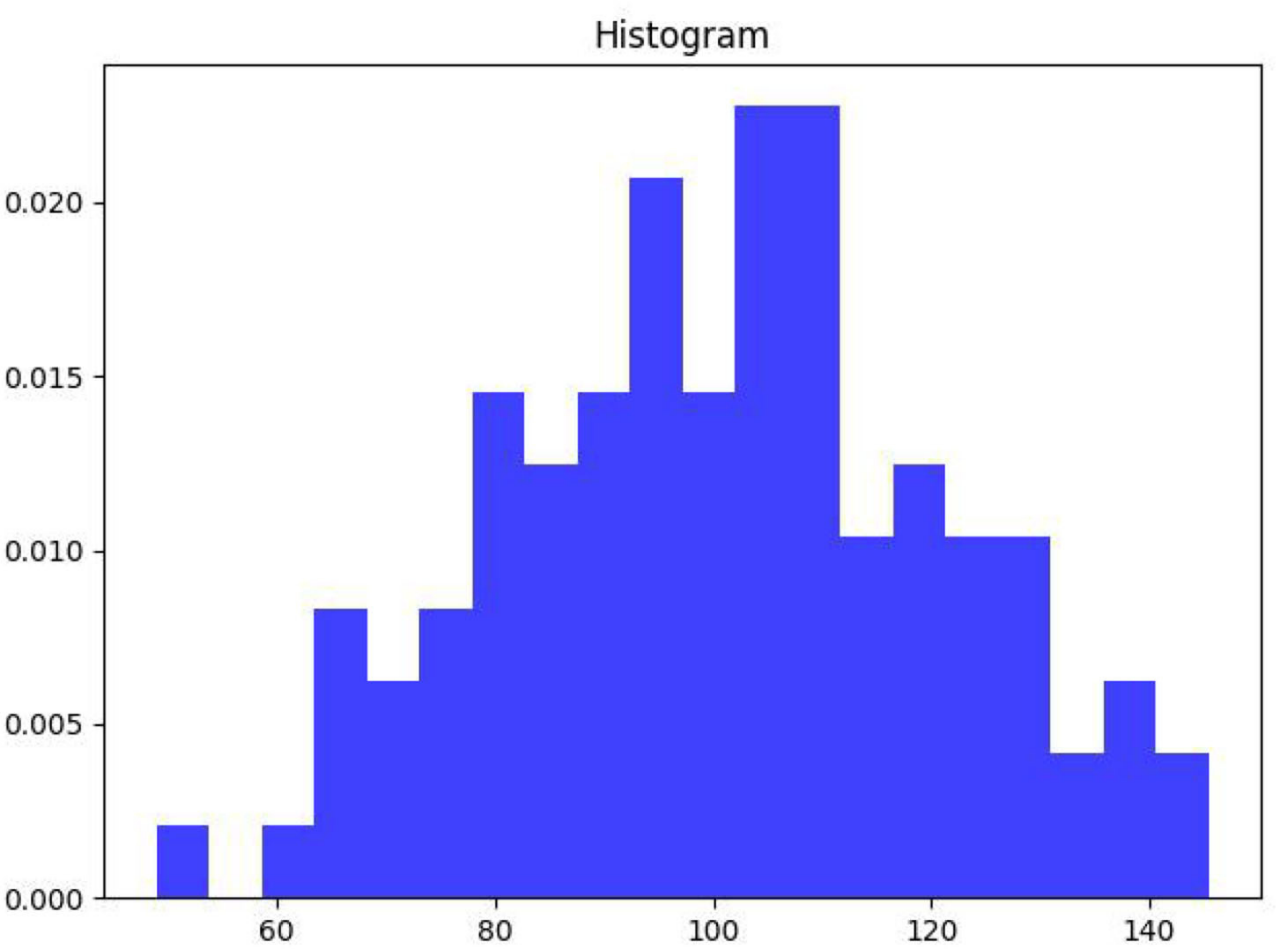
Histogram of word vectors.

As can be seen from Figure 8, the word vector histogram of this paper's scheme, an exact graphical representation of the distribution of the value data. The range of values is segmented, i.e., the entire range of values is divided into a series of intervals, and then how many values are counted in each interval. The values are usually specified as consecutive, non-overlapping intervals of variables. Intervals must be adjacent and usually (but not necessarily) of equal size.

### Recognition of Segmentation Effects

**Figure 9:**
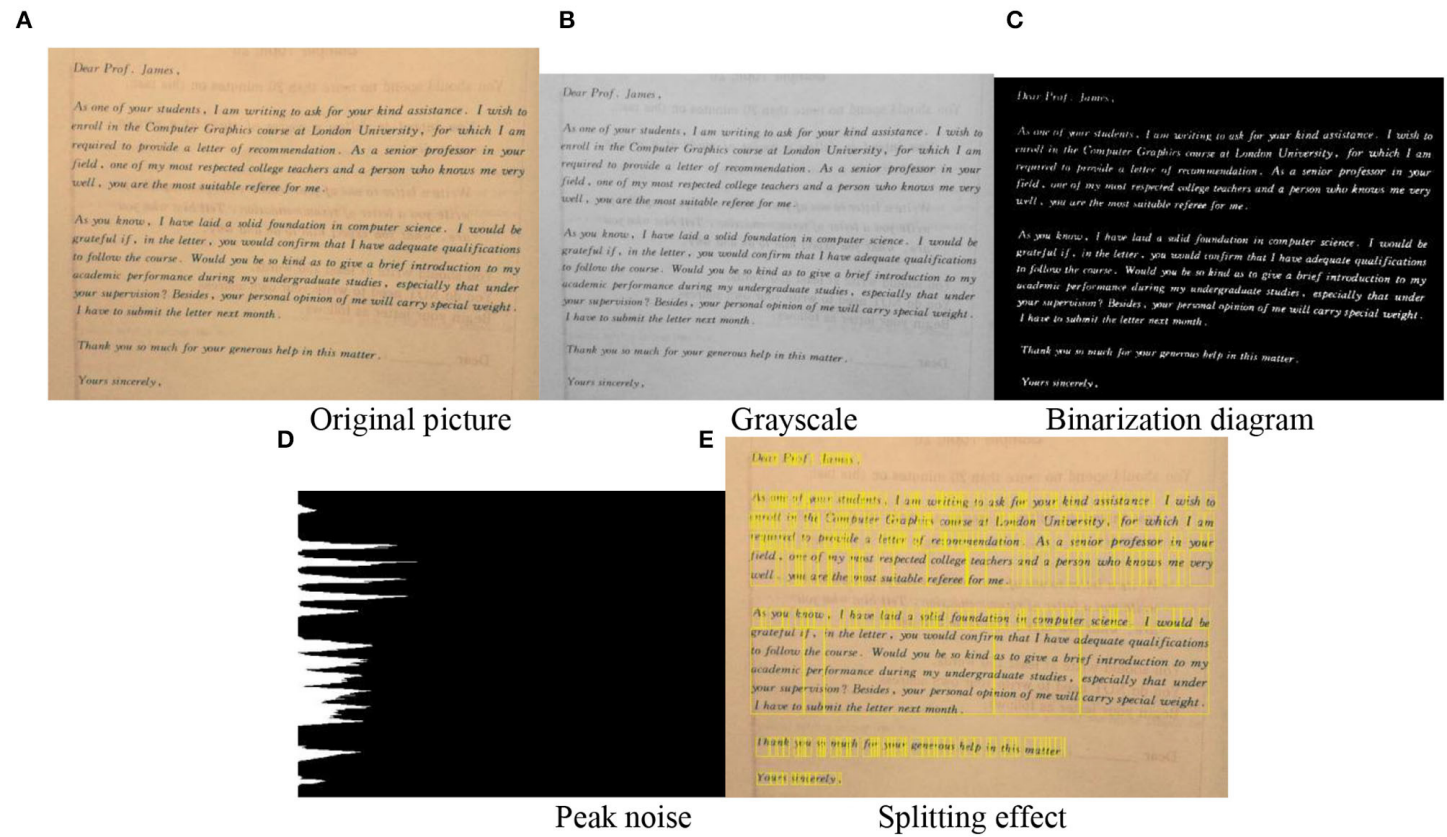
The semantic segmentation process of English units in this model. **(A)** Original picture. **(B)** Grayscale. **(C)** Binarization diagram. **(D)** Peak noise. **(E)** Splitting effect.

The semantic segmentation process of English units in this model is shown in Figure 9, where Figure 9A shows the original image and Figure 9E shows the segmentation effect on English words. It is thought that the pixels in the image with gray scale values in the same class belong to the same object. Since it is a direct application of the gray scale characteristics of the image, the calculation is convenient and concise, and the applicability is strong. Obviously, the key and difficulty of the threshold segmentation approach is how to obtain a suitable threshold value. The threshold setting in Figure 9B is vulnerable to noise and luminance. The approaches in recent years are: the approach of selecting the threshold value with the maximum correlation criterion, the approach based on the image topology stable state, the Yager measure minimization approach, the gray scale co-generation matrix approach, the variance method, the entropy method, the peak and valley analysis method, etc. Figure 9C shows several algorithms that are more successful in improving the traditional shareholding method. In more cases, the selection of thresholds will be a combination of 2 or more approaches, which are also a trend in the development of image segmentation.

## Conclusions

Analyzing English words under the synergistic effect of different modalities, students improve the motivation and effectiveness of word learning, but there are still some problems. In this paper, we construct a stepped network framework based on the Deeplab V3+ network, retain the inflated convolution and code-decoder structures in the original network, and replace the original non-linear activation function ReLU with a more effective visual activation function FReLU by improving the spatial pooling determinant module. Experimental results on the Englishhnd dataset show that the improved network results on the Englishhnd dataset show that the improved network has high recognition accuracy for English characters.

## Data Availability Statement

The raw data supporting the conclusions of this article will be made available by the authors, without undue reservation.

## Author Contributions

LP was responsible for designing the framework of the entire manuscript, from topic selection to solution to experimental verification.

## Conflict of Interest

The author declares that the research was conducted in the absence of any commercial or financial relationships that could be construed as a potential conflict of interest.

## Publisher's Note

All claims expressed in this article are solely those of the authors and do not necessarily represent those of their affiliated organizations, or those of the publisher, the editors and the reviewers. Any product that may be evaluated in this article, or claim that may be made by its manufacturer, is not guaranteed or endorsed by the publisher.

## References

[B1] Cameron L. (2002). Measuring vocabulary size in English as an additional language. Lang. Teach. Res. 6, 145–173. 10.1191/1362168802lr103oa30558314

[B2] Chen Hsieh J. S. Wu W. C. V. Marek M. W. (2017). Using the flipped classroom to enhance EFL learning. Comput. Assist. Lang. Learn. 30, 1–21. 10.1080/09588221.2015.1111910

[B3] Chen M. Wu J. Liu L. Zhao W. Tian F. Shen Q.. (2021). DR-Net: An improved network for building extraction from high resolution remote sensing image. Remote Sens. 13:294. 10.3390/rs13020294

[B4] Dai Y. Huang Z. Gao Y. Xu Y. Chen K. Guo J.. (2018). “Fused text segmentation networks for multi-oriented scene text detection,” in 2018 24th International Conference on Pattern Recognition (ICPR) (Beijing: IEEE), 3604–3609. 10.1109/ICPR.2018.8546066

[B5] Di Wu C. Z. Ji L. Ran R. Wu H. Xu Y. (2021). Forest fire recognition based on feature extraction from multi-view images. Traitement du Signal 38, 775–783. 10.18280/ts.380324

[B6] Duman G. Orhon G. Gedik N. (2015). Research trends in mobile assisted language learning from 2000 to 2012. Recall 27, 197–216. 10.1017/S0958344014000287

[B7] Gu S. Zhang F. (2020). Applicable scene text detection based on semantic segmentation. J. Phys. Conf. Ser. 1631, 012080. 10.1088/1742-6596/1631/1/012080

[B8] Hessamy G. Ghaderi E. (2014). The role of dynamic assessment in the vocabulary learning of Iranian EFL learners. Proc. Soc. Behav. Sci. 98, 645–652. 10.1016/j.sbspro.2014.03.463

[B9] Huang Y. M. Chiu P. S. Liu T. C. Chen T. S. (2011). The design and implementation of a meaningful learning-based evaluation method for ubiquitous learning. Comput. Educ. 57, 2291–2302. 10.1016/j.compedu.2011.05.023

[B10] Huang Z. Zhong Z. Sun L. Huo Q. (2019). “Mask R-CNN with pyramid attention network for scene text detection,” in 2019 IEEE Winter Conference on Applications of Computer Vision (WACV) (Waikoloa, HI: IEEE), 764–772. 10.1109/WACV.2019.00086

[B11] Laufer B. (2006). Comparing focus on form and focus on forms in second-language vocabulary learning. Can. Modern Lang. Rev. 63, 149–166. 10.3138/cmlr.63.1.149

[B12] Liu J. Geng Y. Zhao J. Zhang K. Li W. (2021). Image semantic segmentation use multiple-threshold probabilistic R-CNN with feature fusion. Symmetry 13, 207. 10.3390/sym13020207

[B13] Liu R. Mi L. Chen Z. (2020). AFNet: adaptive fusion network for remote sensing image semantic segmentation. IEEE Trans. Geosci. Remote Sens. 60, 1–16. 10.1109/TGRS.2020.3035561

[B14] Liu W. (2021). Real-time obstacle detection based on image semantic segmentation and fusion network. Traitement du Signal 38, 443–449. 10.18280/ts.38022333322029

[B15] Liu Y. Zhang D. Lu G. Ma W. Y. (2007). A survey of content-based image retrieval with high-level semantics. Pattern Recogn. 40, 262–282. 10.1016/j.patcog.2006.04.045

[B16] Lyu P. Liao M. Yao C. Wu W. Bai X. (2018). “Mask textspotter: an end-to-end trainable neural network for spotting text with arbitrary shapes,” in Proceedings of the European Conference on Computer Vision (ECCV) (Munich), 67–83. 10.1007/978-3-030-01264-9_5

[B17] Radwan A. Huq K. M. S. Mumtaz S. Tsang K. F. Rodriguez J. (2016). Low-cost on-demand C-RAN based mobile small-cells. IEEE Access 4, 2331–2339. 10.1109/ACCESS.2016.2563518

[B18] Saalbach A. Lange O. Nattkemper T. Meyer-Baese A. (2009). On the application of (topographic) independent and tree-dependent component analysis for the examination of DCE-MRI data. Biomed. Signal Process. Control 4, 247–253. 10.1016/j.bspc.2009.03.010 20689662 PMC2916199

[B19] Saghezchi F. B. Radwan A. Rodriguez J. Dagiuklas T. (2013). Coalition formation game toward green mobile terminals in heterogeneous wireless networks. IEEE Wireless Commun. 20, 85–91. 10.1109/MWC.2013.6664478

[B20] Sanonguthai S. (2011). Teaching IELTS writing module through English debate: a case study in Thailand. Lang. Test. Asia 1, 1–61. 10.1186/2229-0443-1-4-39

[B21] Shin H. Ahn B. Bae D. (2016). English vocabulary learning through metacognitive memory strategy and vocabulary testing. J. Modern Brit. Am. Lang. Lit. 34, 121–149. 10.21084/jmball.2016.02.34.1.121

[B22] Sung Y. T. Chang K. E. Liu T. C. (2016). The effects of integrating mobile devices with teaching and learning on students' learning performance: a meta-analysis and research synthesis. Comput. Educ. 94, 252–275. 10.1016/j.compedu.2015.11.008

[B23] Tian Z. Shu M. Lyu P. Li R. Zhou C. Shen X.. (2019). “Learning shape-aware embedding for scene text detection,” in Proceedings of the IEEE/CVF Conference on Computer Vision and Pattern Recognition (Long Beach, CA), 4234–4243. 10.1109/CVPR.2019.00436

[B24] Wu J. Chen B. (2020). English vocabulary online teaching based on machine learning recognition and target visual detection. J. Intell. Fuzzy Syst. 39, 1745–1756. 10.3233/JIFS-179948

[B25] Xie E. Zang Y. Shao S. Yu G. Yao C. Li G. (2019). “Scene text detection with supervised pyramid context network,” in Proceedings of the AAAI Conference on Artificial Intelligence (Hawaii), 9038–9045. 10.1609/aaai.v33i01.33019038

[B26] Xie T. Zhang C. Zhang Z. Yang K. (2018). Utilizing active sensor nodes in smart environments for optimal communication coverage. IEEE Access 7, 11338–11348. 10.1109/ACCESS.2018.2889717

[B27] Xue Y. Geng H. Zhang H. Xue Z. Xu G. (2018). Semantic segmentation based on fusion of features and classifiers. Multimedia Tools Appl. 77, 22199–22211. 10.1007/s11042-018-5858-z

[B28] Yin M. (2021). Research on English vocabulary classification based on computer deep learning. J. Phys. Conf. Ser. 1992, 022074. 10.1088/1742-6596/1992/2/022074

[B29] Zhang C. Liu X. (2021). Feature extraction of ancient Chinese characters based on deep convolution neural network and big data analysis. Comput. Intell. Neurosci. 2021, 1–10. 10.1155/2021/2491116 34504520 PMC8423538

[B30] Zhang C. Xie T. Yang K. Ma H. Xie Y. Xu Y.. (2019). Positioning optimisation based on particle quality prediction in wireless sensor networks. IET Netw. 8, 107–113. 10.1049/iet-net.2018.5072

[B31] Zhang R. Li G. Li M. Wang L. (2018). Fusion of images and point clouds for the semantic segmentation of large-scale 3D scenes based on deep learning. ISPRS J. Photogramm. Remote Sens. 143, 85–96. 10.1016/j.isprsjprs.2018.04.022

[B32] Zhang Z. Zhang C. Li M. Xie T. (2020). Target positioning based on particle centroid drift in large-scale WSNs. IEEE Access 8, 127709–127719. 10.1109/ACCESS.2020.3008373

[B33] Zhao W. Wang B. Coniam D. Xie B. (2017). Calibrating the CEFR against the China Standards of English for College English vocabulary education in China. Lang. Test. Asia 7, 1–18. 10.1186/s40468-017-0036-1

[B34] Zhou H. Fang Z. Gao Y. Huang B. Zhong C. Shang R. (2020). Feature fusion network based on attention mechanism for 3D semantic segmentation of point clouds. Pattern Recogn. Lett. 133, 327–333. 10.1016/j.patrec.2020.03.021

